# Targeting Drp1 in Cerebral Ischemia–Reperfusion Injury: Mechanisms and Therapeutic Implications

**DOI:** 10.1111/cns.70590

**Published:** 2025-08-28

**Authors:** Mengnan Liu, Zhixue Yin, Binru Li, Ji Qiu, Dechou Zhang, Raoqiong Wang, Xue Bai, Li Chen

**Affiliations:** ^1^ Department of Cardiovascular Medicine, the Affiliated Traditional Chinese Medicine Hospital Southwest Medical University Luzhou Sichuan China; ^2^ Southwest Medical University Luzhou Sichuan China; ^3^ Department of Neurology Minzu Hospital of Guangxi Zhuang Autonomous Region Nanning Guangxi China; ^4^ Luhuo County Niba Township Health Center Luhuo Sichuan China; ^5^ Department of Neurology, the Affiliated Traditional Chinese Medicine Hospital Southwest Medical University Luzhou Sichuan China

**Keywords:** cerebral ischemia–reperfusion injury (CIRI), Drp1, mitochondrial dynamics, neuroprotection, oxidative stress

## Abstract

**Background:**

Cerebral ischemia‐reperfusion injury (CIRI) arises after blood flow restoration in stroke, where reperfusion paradoxically triggers mitochondrial dysfunction, apoptosis, inflammation, and oxidative stress. Dynamin‐related protein 1 (Drp1), a regulator of mitochondrial fission, amplifies these cascades by promoting apoptosis, inflammatory signaling, and calcium imbalance.

**Methods:**

This review synthesizes recent studies on Drp1 in CIRI, focusing on its regulatory roles in mitochondrial dynamics and neuronal injury, and evaluating therapeutic strategies through pharmacological and genetic modulation.

**Results:**

Evidence shows Drp1 inhibition mitigates CIRI in preclinical models by restoring mitochondrial homeostasis, reducing oxidative stress, and improving neuronal survival. Promising interventions include selective inhibitors and genetic approaches, though challenges remain regarding drug specificity, delivery efficiency, and long‐term safety.

**Conclusion:**

Drp1 is central to CIRI pathology and represents a promising therapeutic target. Future work should prioritize advanced delivery systems and safer, more selective Drp1 modulators to enable clinical translation.

## Graphical abstract

This review highlights the central role of dynamin‐related protein 1 (Drp1) in cerebral ischemia–reperfusion injury (CIRI), emphasizing its involvement in mitochondrial dysfunction, oxidative stress, and neuronal injury. Drp1 is presented as a promising therapeutic target for CIRI, with evidence suggesting that modulating its activity can alleviate mitochondrial damage and reduce neuronal loss. The review also discusses the challenges in translating Drp1‐targeted therapies from preclinical models to clinical settings, particularly issues related to drug specificity, delivery, and safety.

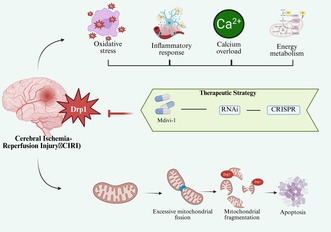

**Abbreviations**
AMPK: AMP‐activated protein kinase
ATP: adenosine triphosphate
BBB: blood–brain barrier
CIRI: cerebral ischemia–reperfusion injury
DAMPs: molecular patterns
Drp1: dynamin‐related protein 1
ETC: electron transport chain
GED: GTPase effector domain
GTP: guanosine triphosphate
Mdivi‐1: mitochondrial division Inhibitor 1
Mff: mitochondrial fission factor
Mfn: mitofusins
mPTP: mitochondrial permeability Transition Pore
mRNA: messenger RNA
mtDNA: mitochondrial DNA
NAC: N‐acetylcysteine
NLRP3: NOD‐like receptor pyrin 3
NSAIDs: nonsteroidal anti‐inflammatory drugs
OPA1: optic atrophy protein 1
PQQ: pyrroloquinoline quinone
PTMs: post‐translational modifications
RNAi: RNA interference
ROS: reactive oxygen species
siRNA: small interfering RNA
SUMO: small ubiquitin‐like modifier
ΔΨm: mitochondrial membrane potential

## 1 Introduction

Cerebral ischemia–reperfusion injury (CIRI) is a major complication of ischemic stroke, occurring when blood flow is restored to previously ischemic brain tissue. While reperfusion is critical for delivering oxygen and nutrients, it paradoxically initiates a series of detrimental biochemical and cellular processes that exacerbate brain injury. These processes include mitochondrial dysfunction, oxidative stress, calcium dysregulation, inflammation, and apoptosis, ultimately leading to neuronal damage and cell death. The multifactorial nature of CIRI poses significant challenges for effective treatment, as current therapeutic strategies often fall short in mitigating the adverse effects of reperfusion injury [1].

Recent research has highlighted the pivotal role of Drp1 in the pathogenesis of CIRI. As a key regulator of mitochondrial fission, Drp1‐driven mitochondrial fragmentation is a central feature of ischemia–reperfusion injury. This fragmentation contributes to excessive production of reactive oxygen species (ROS), impaired ATP synthesis, and the release of pro‐apoptotic factors. Furthermore, Drp1 activity is regulated by various post‐translational modifications (PTMs), including phosphorylation, SUMOylation, and acetylation, which enhance its pathological effects under ischemic conditions. Dysregulated Drp1 activity has been implicated in several pathological mechanisms, such as calcium homeostasis disruption, NLRP3 inflammasome activation, and neuronal apoptosis, suggesting that Drp1 is a promising therapeutic target for reducing ischemia–reperfusion‐induced neuronal damage [2].

Despite advancements in understanding Drp1's role in CIRI, comprehensive therapeutic strategies targeting Drp1 remain limited. Current research has explored pharmacological inhibitors, gene‐based interventions, and combination therapies aimed at modulating Drp1 activity. However, their clinical translation faces significant challenges, including poor specificity, limited blood–brain barrier penetration, and potential off‐target effects. Among these strategies, pharmacological inhibitors such as Mitochondrial Division Inhibitor 1 (Mdivi‐1) have demonstrated promising neuroprotective effects in preclinical models by reducing Drp1 activity, stabilizing mitochondrial function, and mitigating oxidative damage [3]. Recent studies have also focused on developing more selective inhibitors targeting Drp1's GTPase domain and post‐translational modification sites, which may enhance their therapeutic efficacy and safety profile [4].

Given Drp1's complex and multifaceted role in CIRI, a thorough understanding of its regulatory mechanisms and interactions with other cellular pathways is essential for developing effective therapeutic strategies. This review aims to provide a comprehensive overview of Drp1's involvement in CIRI, discuss current and emerging therapeutic strategies targeting Drp1, and highlight future research directions.

## 2 Mitochondrial Dysregulation Mechanisms in CIRI

CIRI is a complex pathological process involving multiple interrelated mechanisms, with mitochondrial dysfunction being a key contributor.

### 2.1 Mitochondrial Dysfunction and Oxidative Stress in CIRI

Mitochondria are critical for cellular energy production and maintaining homeostasis. In CIRI, mitochondrial dysfunction is a central feature. During reperfusion, the sudden influx of oxygen triggers the production of ROS, which causes oxidative damage to mitochondrial DNA, proteins, and lipids. This oxidative damage reduces mitochondrial membrane potential (ΔΨm), disrupts ATP production, and increases neuronal vulnerability to injury. The resulting oxidative stress also accelerates ROS production in a feedback loop, further contributing to mitochondrial dysfunction. This amplification of oxidative stress leads to cellular damage and accelerates neuronal death, making oxidative stress a key therapeutic target for mitigating CIRI‐induced damage [5].

### 2.2 Mitochondrial Fission and Fusion in CIRI

The balance between mitochondrial fission and fusion is essential for maintaining mitochondrial function and integrity. In CIRI, excessive mitochondrial fission is driven by oxidative stress, calcium overload, and inflammatory cytokines. This results in mitochondrial fragmentation, which disrupts mitochondrial function and amplifies oxidative damage. Mitochondrial fragmentation leads to the formation of smaller, dysfunctional mitochondrial units that generate higher levels of ROS, which further exacerbate the damage to neuronal cells. This imbalance in mitochondrial dynamics significantly contributes to CIRI and promotes cell death pathways such as apoptosis [6].

### 2.3 Calcium Dysregulation and Mitochondrial Calcium Overload in CIRI

Calcium dysregulation plays a crucial role in CIRI. During reperfusion, calcium ions accumulate in mitochondria, triggering mitochondrial calcium overload. This overload leads to the opening of the mitochondrial permeability transition pore (mPTP), resulting in the loss of ΔΨm and mitochondrial membrane rupture. The opening of mPTP exacerbates oxidative stress, activates apoptosis, and induces cell death. Additionally, calcium overload activates calcineurin, a calcium‐dependent phosphatase, which further contributes to mitochondrial fragmentation and enhances the pathological mitochondrial fission cycle, amplifying the injury response and neuronal damage in CIRI [7].

### 2.4 Mitophagy Impairment in CIRI

Mitophagy is the selective autophagic degradation of damaged mitochondria and is essential for maintaining mitochondrial quality control. However, during CIRI, oxidative stress and inflammation impair mitophagy, preventing the clearance of dysfunctional mitochondria. These damaged mitochondria generate excessive ROS and release damage‐associated molecular patterns (DAMPs), such as mitochondrial DNA, which activate inflammatory pathways, including the formation of the NLRP3 inflammasome. Impaired mitophagy allows dysfunctional mitochondria to persist, increasing ROS production, further mitochondrial damage, and inflammation, thereby exacerbating neuronal injury and contributing to the pathogenesis of CIRI [8].

## 3 Structure, Regulation Mechanisms, and Function of Drp1

### 3.1 Structure and Regulatory Mechanisms of Drp1

Drp1, a key GTPase involved in mitochondrial fission, has a structure comprising the N‐terminal GTPase domain, middle domain, B‐insert (variable domain), and C‐terminal GTPase Effector Domain (GED), all of which are integral to mitochondrial dynamics [9]. The GTPase domain facilitates Drp1's GTP hydrolysis, enabling its oligomerization into ring‐like structures around mitochondria, a process essential for fission. Phosphorylation at Ser616 activates Drp1 to promote fission, while phosphorylation at Ser637 stabilizes it in an elongated state. During CIRI, calcineurin‐mediated dephosphorylation of Ser637, triggered by calcium influx, recruits Drp1 to the mitochondria, intensifying mitochondrial fragmentation [10].

The middle domain contributes to Drp1 oligomer stability, providing structural flexibility that allows the protein to adapt to various mitochondrial curvatures through the B‐insert. The C‐terminal GED anchors Drp1 by binding to adaptor proteins such as Fis1, Mff, Mid49, and Mid51, which are critical for positioning Drp1 at fission sites. Mutations or structural changes in these domains impair Drp1 binding, leading to mitochondrial dysfunction, particularly in the context of CIRI [11, 12].

Drp1's function is finely regulated by PTMs, including phosphorylation, SUMOylation, ubiquitination, and acetylation. Phosphorylation at Ser515, Ser579, and Ser585 enhances Drp1's mitochondrial recruitment and fission activity under oxidative and inflammatory stress. SUMOylation at Lys680 stabilizes Drp1 on the mitochondrial membrane, amplifying its activity by preventing proteasomal degradation and intensifying fragmentation during ischemic stress [13, 14]. Palmitoylation further strengthens Drp1's membrane association, ensuring sustained activity during CIRI, while ubiquitination regulates Drp1 turnover by promoting either degradation or stabilization. Acetylation modulates Drp1's fission activity, with deacetylation by SIRT3 under stress enhancing its function and contributing to cellular quality control [15, 16].

Drp1 regulation extends through several key signaling pathways. In CIRI, calcium signaling activates calcineurin, which dephosphorylates Ser637, facilitating Drp1 recruitment to the mitochondria and initiating fission [17, 18]. ROS signaling further elevates Drp1 translocation, creating a feedback loop that amplifies mitochondrial fragmentation and exacerbates injury [19]. Apoptotic signaling involves interactions with pro‐apoptotic proteins like Bax and Bak, leading to mitochondrial membrane permeabilization and apoptosis, crucial in ischemic neuronal damage [20]. Under energy stress, the AMPK pathway enhances Drp1's interaction with Mff, aligning mitochondrial fission with cellular energy demands [21]. In contrast, the PI3K/Akt pathway reduces mitochondrial fragmentation by inhibiting Drp1, offering neuroprotection in CIRI. At mitochondria‐ER contact sites (MERCs), Drp1 interacts with ER proteins like INF2, facilitating calcium transfer from the ER to mitochondria, which intensifies damage under ischemic conditions [22].

### 3.2 Drp1's Physiological Roles

Drp1 is a cytosolic large GTPase that plays an essential role in mitochondrial fission, a highly regulated process required for mitochondrial quality control and cellular homeostasis [23]. Under physiological conditions, Drp1 is recruited from the cytosol to the outer mitochondrial membrane, where it oligomerizes and interacts with adaptor proteins such as Fis1, Mff, Mid49, and Mid51. This action facilitates the scission of mitochondria, allowing for proper mitochondrial distribution, morphological remodeling, and the elimination of damaged mitochondria via mitophagy [24, 25].

Drp1‐mediated mitochondrial fission is crucial for several key cellular processes, including ATP production through oxidative phosphorylation, buffering of cytosolic calcium, control of ROS generation, and the initiation of intrinsic apoptotic signaling. In particular, neurons depend heavily on Drp1‐regulated mitochondrial dynamics due to their high metabolic demands and vulnerability to bioenergetic disruption [26]. Drp1 also functions as a priming factor in mitophagy, enabling selective degradation of dysfunctional mitochondria through the PINK1/Parkin pathway, thereby preserving mitochondrial integrity [27].

Although Drp1 plays a critical role in all brain cells, its function varies across different cell types, particularly in neurons, astrocytes, and microglia. In neurons, Drp1 regulates mitochondrial morphology and distribution to meet the high energy demands and protects neurons from damage by modulating mitochondrial fission and supporting autophagy processes [28]. In contrast, in astrocytes, Drp1 primarily supports neuronal metabolism by maintaining mitochondrial function and, under pathological conditions, regulates neuroinflammatory responses, thereby contributing to neuroprotection [29]. In microglia, Drp1 regulates mitochondrial dynamics to support immune function, but in pathological conditions such as cerebral ischemia, excessive activation of Drp1 can exacerbate neuroinflammation and contribute to brain damage. Inhibiting the overactivation of Drp1 in microglia may help alleviate this excessive response and protect neurons [30].

## 4 The Role of Drp1 in CIRI

CIRI is a complex pathological process that arises following the restoration of blood flow to the brain after an ischemic stroke. While reperfusion is essential for delivering oxygen and nutrients to ischemic brain tissue, it simultaneously triggers a cascade of harmful reactions, including mitochondrial dysfunction, oxidative stress, calcium dysregulation, inflammation, and apoptosis [31, 32]. Drp1, a key regulator of mitochondrial fission, plays a significant role in these pathological processes.

### 4.1 Drp1's Role in Normal Brain Function

In normal, Drp1 is essential for maintaining mitochondrial dynamics by regulating the balance between mitochondrial fission and fusion. This balance ensures proper mitochondrial function, facilitates the removal of damaged mitochondria through mitophagy, and supports cellular energy production via ATP synthesis [33]. Drp1 activity is tightly regulated to adapt to fluctuating metabolic demands [34].

### 4.2 The Multifaceted Role of Drp1 in CIRI: Mechanistic Insights

**FIGURE 1:**
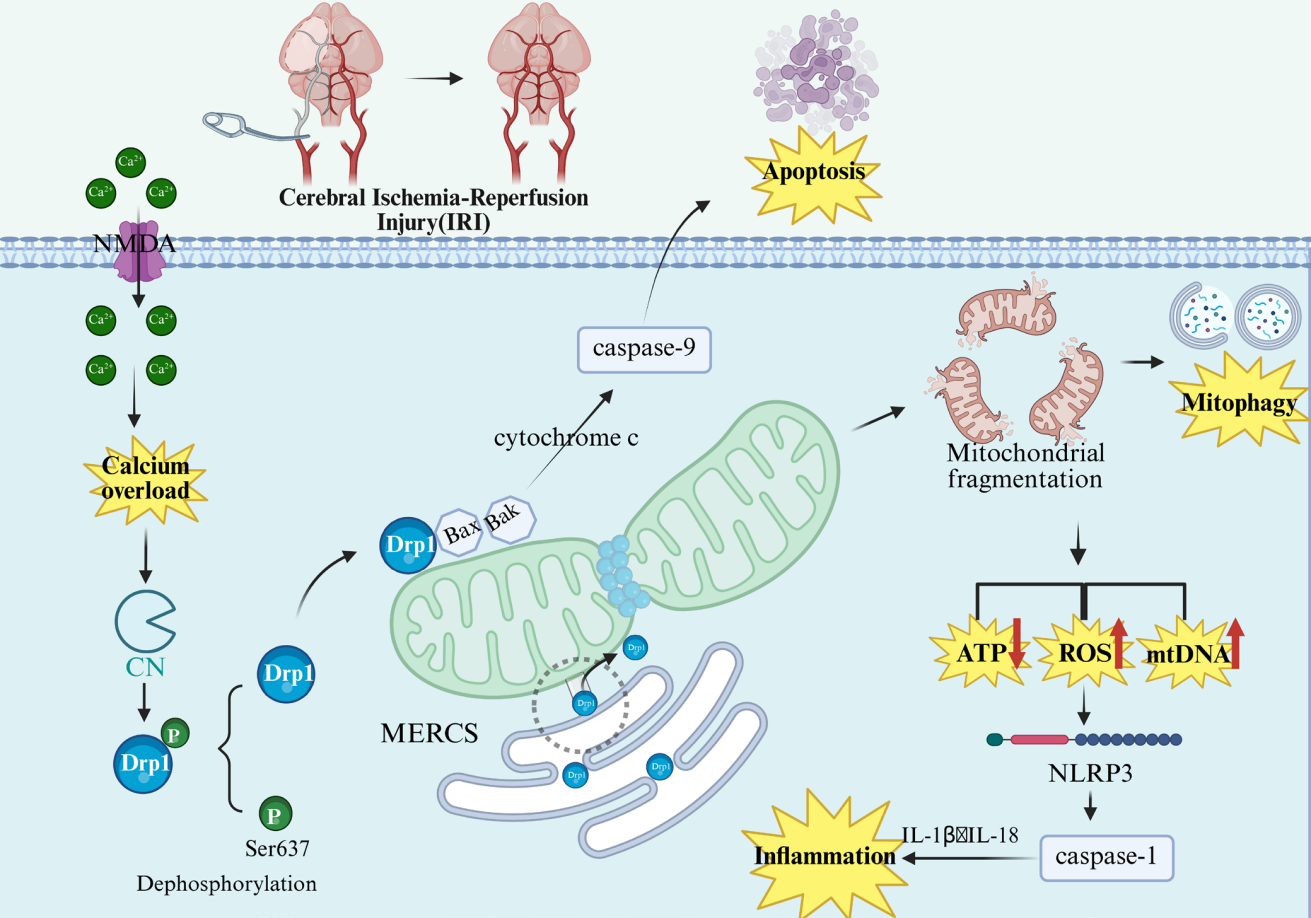
Pathological Mechanisms of Drp1 in cerebral ischemia–reperfusion injury (CIRI). Drp1 plays a key role in CIRI. When overactivated, it causes excessive mitochondrial fission, reduces mitochondrial function, and increases ROS production, leading to cellular damage. Drp1 also affects calcium balance, oxidative stress, apoptosis, and inflammation, contributing to neuronal injury.

During CIRI, Drp1 is excessively activated in response to various cellular stressors, resulting in pathological mitochondrial fission. This overactivation leads to mitochondrial fragmentation, loss of ΔΨm, and impaired ATP production [35, 36]. The ensuing mitochondrial dysfunction compromises mitochondrial integrity, triggers the release of pro‐apoptotic factors, and activates apoptotic signaling pathways, which ultimately contribute to neuronal death and exacerbate ischemic injury. Additionally, Drp1 dysregulation significantly influences other pathological mechanisms, including oxidative stress, calcium overload, and inflammation, further compounding the damage associated with CIRI [37]. These mechanisms are complex and involve numerous molecules and signaling pathways, as depicted in Figure 1, which illustrates the regulatory role of Drp1 in mediating the mechanisms of brain ischemia–reperfusion injury.

#### 4.2.1 Drp1's Mechanistic Role in Mitochondrial Dysfunction During CIRI

During CIRI, overactivation of Drp1 disrupts the balance between mitochondrial fission and fusion, leading to pathological mitochondrial fragmentation. Under normal conditions, mitochondrial fission and fusion cycles are crucial for maintaining cellular quality control, ATP production, and overall mitochondrial function. However, CIRI introduces stressors such as elevated ROS and calcium overload, which excessively activate Drp1, shifting mitochondrial dynamics toward excessive fission. This pathological shift reduces ΔΨm, impairing ETC function, decreasing ATP synthesis, and compromising the energy supply—effects that are particularly detrimental to energy‐demanding neuronal cells [38].

Fragmented mitochondria become increasingly vulnerable to ROS leakage due to ETC inefficiencies. Accumulated ROS causes oxidative damage to mitochondrial DNA, lipids, and proteins, further impairing mitochondrial function. This oxidative stress perpetuates Drp1 overactivation, creating a feedback loop that exacerbates mitochondrial fragmentation and dysfunction. The resulting vicious cycle of ROS‐driven Drp1 activation amplifies mitochondrial and cellular damage, driving the pathological progression of CIRI and contributing to substantial neuronal injury in the ischemic brain [39].

#### 4.2.2 Drp1 and Energy Metabolism Dysregulation

Energy metabolism dysregulation is a critical factor in CIRI, significantly impacting neuronal survival. Drp1, a key regulator of mitochondrial fission, normally balances fission and fusion to maintain mitochondrial network integrity, supporting ATP synthesis through oxidative phosphorylation (OXPHOS). However, during CIRI, Drp1 overactivation disrupts this balance, leading to excessive mitochondrial fragmentation and impaired ATP production, which compromises cellular energy supply, especially in energy‐demanding neurons. Fragmented mitochondria reduce ETC efficiency, resulting in ATP depletion and destabilizing neuronal function, disrupting processes essential for ion homeostasis and neurotransmitter release [2, 40].

Moreover, fragmented mitochondria generate increased ROS, causing damage to mitochondrial DNA (mtDNA) and OXPHOS‐related proteins, thereby exacerbating neuronal energy deficits. Experimental studies indicate that Drp1 inhibition, using agents like Mdivi‐1, preserves mitochondrial structure, stabilizes ATP production, and reduces ROS levels, helping to mitigate neuronal injury by preserving mitochondrial integrity and cellular energy balance. By preventing ATP depletion and protecting mitochondrial function, targeting Drp1 shows promise for enhancing neuronal resilience in CIRI, underscoring its therapeutic potential to counter energy crises and support neuronal repair [41].

#### 4.2.3 Drp1 and Oxidative Stress

Oxidative stress is a critical contributor to CIRI, driven primarily by increased ROS production from dysfunctional mitochondria. Under normal conditions, Drp1‐mediated fission plays a protective role by isolating damaged mitochondria for degradation, thereby controlling ROS levels. However, during CIRI, excessive Drp1 activation leads to persistent mitochondrial fragmentation, resulting in elevated ROS levels and intensified oxidative stress [42, 43]. Fragmented mitochondria with impaired ETC function exacerbate ROS production, establishing a damaging feedback loop where ROS further activate Drp1, compounding mitochondrial and cellular damage [44].

Inhibiting Drp1 with agents such as Mdivi‐1 has been shown to reduce ROS levels by stabilizing mitochondrial structure, restoring ETC function, and limiting oxidative damage. Additionally, Drp1 inhibition prevents the release of mtDNA, which activates inflammatory pathways and exacerbates cellular injury in CIRI. These findings highlight Drp1's dual role in modulating oxidative stress and inflammation, underscoring its potential as a therapeutic target in mitigating CIRI‐induced damage [45, 46].

#### 4.2.4 Drp1's Role in Apoptosis

In CIRI, apoptosis is a major contributor to neuronal loss, with Drp1 playing a pivotal role in mitochondrial‐mediated apoptotic pathways [47]. Under normal conditions, Drp1 activity is tightly regulated to maintain mitochondrial integrity and support cell survival. However, during CIRI, pathological overactivation of Drp1 leads to excessive mitochondrial fission and mitochondrial outer membrane permeabilization (MOMP), a critical step in apoptosis initiation [48]. Drp1 interacts with pro‐apoptotic proteins Bax and Bak, facilitating pore formation on the mitochondrial membrane, which allows cytochrome c and other apoptogenic factors to enter the cytosol, activating caspase pathways and driving apoptosis [20, 49]. Experimental studies have shown that inhibiting Drp1, either pharmacologically with agents like Mdivi‐1 or through genetic approaches, significantly reduces Bax/Bak association with mitochondria, thereby limiting cytochrome c release and caspase activation [50]. In animal models of CIRI, Drp1 inhibition has been associated with reduced neuronal apoptosis, smaller infarct volumes, and improved neurological outcomes, highlighting its therapeutic potential [51].

Recent findings also reveal that Drp1 overactivation engages apoptosis‐inducing factor (AIF)‐mediated pathways, a caspase‐independent mechanism. During CIRI, excessive Drp1 activity promotes AIF translocation from mitochondria to the nucleus, triggering chromatin condensation and DNA fragmentation. This process further contributes to neuronal damage, even in the presence of caspase inhibition. These observations underscore the dual role of Drp1 in mediating both caspase‐dependent and AIF‐mediated apoptotic pathways, making it a promising therapeutic target for comprehensive neuroprotection in CIRI [52, 53].

#### 4.2.5 Drp1 and Calcium Dysregulation

In CIRI, calcium homeostasis is severely disrupted due to a sudden influx of calcium ions during reperfusion, overwhelming cellular buffering capacities and resulting in mitochondrial calcium overload [54]. Drp1, activated through calcium‐dependent pathways such as calcineurin signaling, plays a pivotal role in this dysregulation. Calcineurin dephosphorylates Drp1 at sites such as Ser637, prompting its translocation to mitochondria and initiating excessive mitochondrial fission. Under high calcium levels, overactivated Drp1 induces mitochondrial fragmentation, destabilizing ΔΨm, impairing ATP production, and reducing mitochondrial calcium‐sequestering capacity. This perpetuates intracellular calcium imbalance, further exacerbating mitochondrial and cellular damage [55, 56]. The resulting mitochondrial calcium overload can also trigger the opening of the mitochondrial mPTP, leading to mitochondrial swelling, further ΔΨm collapse, and cell death [57]. Research has demonstrated that inhibiting Drp1 in CIRI models stabilizes ΔΨm, limits mitochondrial calcium accumulation, and prevents mPTP opening, thereby preserving mitochondrial function under ischemic stress [58]. In addition to its impact on energy metabolism, Drp1‐mediated calcium overload contributes to excitotoxicity. Elevated intracellular calcium activates enzymes such as phospholipases, proteases, and endonucleases, which degrade critical cellular components and drive neuronal apoptosis [59]. By inhibiting Drp1, mitochondrial calcium influx is reduced, attenuating excitotoxic signaling pathways. Experimental models of CIRI have shown that Drp1 inhibition prevents excitotoxic injury, decreases neuronal apoptosis, and enhances recovery after ischemic insult, underscoring the therapeutic potential of targeting Drp1 to manage calcium dysregulation in CIRI [60].

Moreover, Drp1‐mediated calcium overload has been linked to inflammation in CIRI. Persistent mitochondrial calcium overload promotes the release of DAMPs, such as mtDNA, into the cytosol. These DAMPs activate inflammasomes like NLRP3, leading to the release of inflammatory cytokines such as IL‐1β, which further exacerbate neuronal injury [61]. Targeting Drp1 may therefore offer a dual benefit in mitigating both excitotoxic and inflammatory pathways, highlighting its promise as a therapeutic strategy in CIRI.

#### 4.2.6 Drp1's Role in Inflammation

Inflammation is a critical pathological process in CIRI, exacerbating neuronal damage and impeding recovery. Drp1 plays a central role in modulating inflammation by impacting mitochondrial integrity and releasing pro‐inflammatory signals. During CIRI, Drp1‐mediated mitochondrial fragmentation promotes the release of mtDNA and ROS, which act as DAMPs that activate the NLRP3 inflammasome. This activation triggers a cascade involving caspase‐1, leading to the maturation of pro‐inflammatory cytokines such as IL‐1β and IL‐18, which further amplify inflammation and neuronal injury [62, 63]. Research indicates that inhibiting Drp1 reduces mitochondrial fragmentation, limits mtDNA release, and suppresses inflammasome activation, highlighting its potential as a therapeutic target for controlling neuroinflammation in CIRI [64].

Drp1 also influences inflammation through its effects on mitochondrial quality control, particularly mitophagy. Under normal conditions, mitophagy eliminates damaged mitochondria, reducing ROS and DAMP accumulation. However, excessive Drp1 activity during CIRI disrupts mitophagy, resulting in the buildup of dysfunctional mitochondria that continuously release ROS and mtDNA, sustaining inflammation [65]. Studies have shown that Drp1 inhibition restores mitophagy, decreases DAMP levels, and attenuates NLRP3 inflammasome activation. Furthermore, inflammation can create a feedback loop, where pro‐inflammatory cytokines such as TNF‐α upregulate Drp1 expression through NF‐κB and MAPK signaling pathways. This upregulation exacerbates mitochondrial fragmentation and perpetuates inflammation, emphasizing the complex interplay between Drp1 and inflammatory processes in CIRI [66, 67].

#### 4.2.7 Drp1 and Mitophagy

Mitophagy is a critical quality control mechanism that selectively degrades damaged mitochondria, maintaining cellular function and energy balance. Under normal conditions, Drp1‐mediated mitochondrial fission isolates dysfunctional mitochondria, facilitating their removal through mitophagy and supporting mitochondrial and cellular health. However, in CIRI, pathological overactivation of Drp1 disrupts this balance, leading to excessive mitochondrial fragmentation that overwhelms the mitophagic system and exacerbates mitochondrial dysfunction [68, 69].

In CIRI, damaged mitochondria release signals to initiate mitophagy and prevent the spread of dysfunction. Drp1 typically supports this process by segregating compromised mitochondria from the healthy network. However, CIRI‐induced Drp1 hyperactivation results in excessive mitochondrial fragmentation, causing either insufficient clearance of damaged mitochondria or excessive degradation of mitochondrial components. This imbalance reduces ATP production and contributes to neuronal injury. Studies have shown that pharmacological inhibition of Drp1, using agents like Mdivi‐1, restores mitochondrial balance in CIRI by reducing fragmentation, preserving ATP synthesis, and maintaining mitochondrial DNA integrity. Drp1 inhibition has also been shown to enhance interactions with mitophagy‐related proteins such as PINK1 and Parkin, improving the clearance of damaged mitochondria and reducing inflammatory signaling [70, 71].

Overall, Drp1 plays a dual role in mitophagy: while it supports damaged mitochondria removal under normal conditions, its overactivation in CIRI disrupts mitochondrial turnover. Modulating Drp1 to restore mitophagy balance may provide neuroprotection by supporting mitochondrial health, reducing oxidative damage, and aiding cellular recovery following ischemic events.

## 5 Targeting Drp1 as a Therapeutic Strategy for CIRI

**FIGURE 2:**
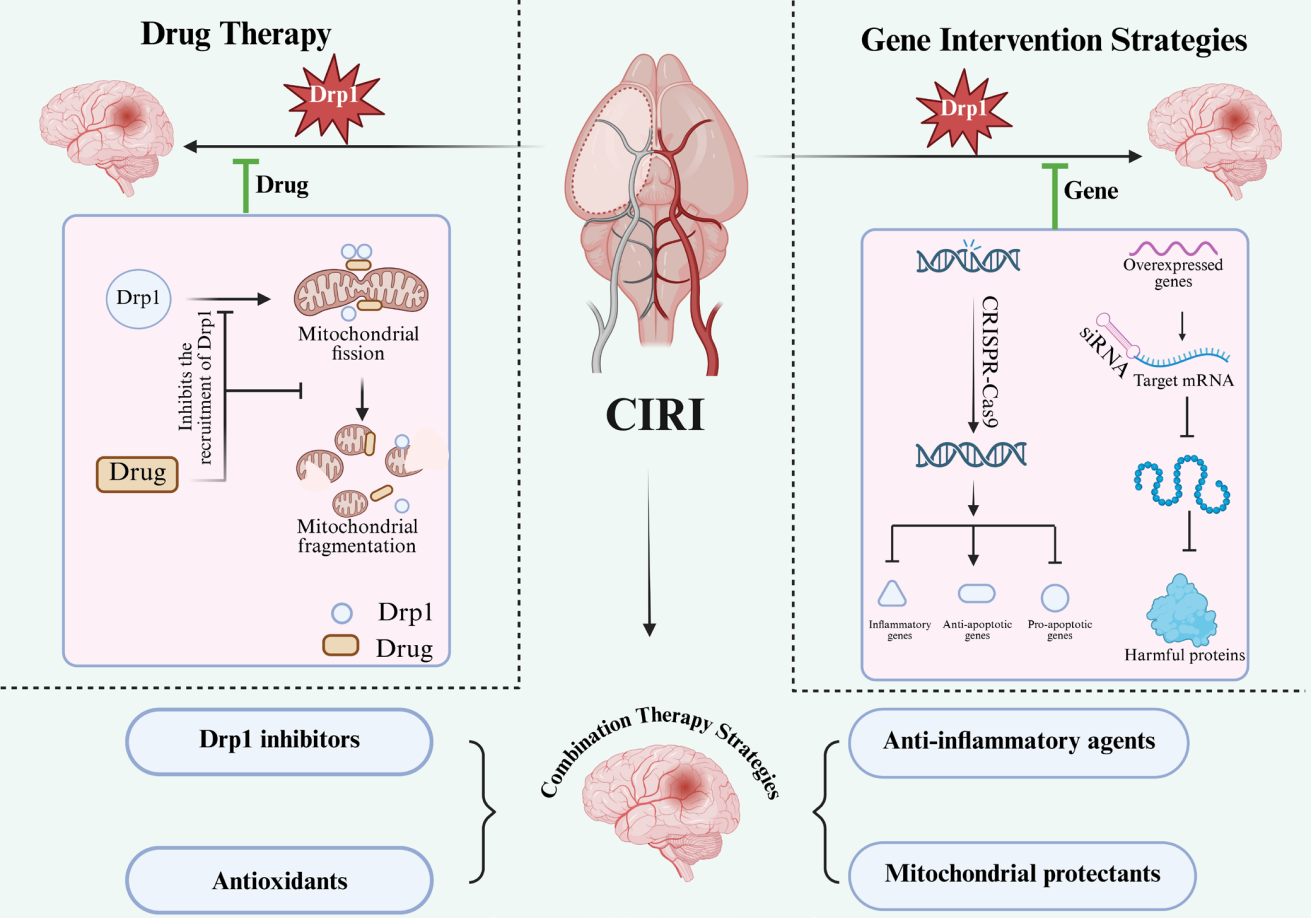
Three Strategies Targeting Drp1 to Alleviate Cerebral Ischemia–Reperfusion Injury: Pharmacological, Gene Intervention, and Combination Therapy Approaches. *Left Panel*: The first strategy involves using Drp1 inhibitors to block mitochondrial fission and protect neurons. *Right Panel*: The second strategy uses gene techniques to reduce Drp1 expression and prevent neuronal damage. *Bottom Panel*: The third strategy combines Drp1 inhibitors with antioxidants and anti‐inflammatory agents to enhance neuroprotection.

Given its central role in the pathogenesis of CIRI, Drp1 has emerged as a promising therapeutic target [72]. It plays a pivotal role in mitochondrial dysfunction, oxidative stress, apoptosis, and calcium overload, all of which contribute to ischemic neuronal injury. Modulating Drp1 activity therapeutically holds potential for preserving mitochondrial function, reducing ROS accumulation, and preventing apoptotic cell death, thereby offering neuroprotection [73]. However, it is crucial to maintain a balance between inhibiting pathological mitochondrial fission and preserving physiological mitochondrial dynamics. While inhibiting Drp1 can effectively reduce excessive mitochondrial fragmentation and cell death, Drp1 also plays a vital role in maintaining normal cellular functions, including energy metabolism, calcium homeostasis, and mitophagy [74]. Therefore, future therapeutic strategies should aim to selectively target pathological fission without disrupting Drp1's normal physiological functions. Recent studies have investigated various strategies for targeting Drp1, including small‐molecule inhibitors, gene silencing techniques, and modulation of PTMs, as illustrated in Figure 2.

### 5.1 Pharmacological Application of Drp1 Inhibitors

Pharmacological inhibition of Drp1 has become one of the most studied strategies for mitigating the harmful effects of CIRI. Mdivi‐1, in particular, has been widely studied for its ability to inhibit Drp1's GTPase activity, preventing pathological mitochondrial fission. Preclinical studies have shown that Mdivi‐1 helps preserve ΔΨm, stabilizes oxidative phosphorylation, and inhibits the release of pro‐apoptotic factors like cytochrome c [75]. By blocking excessive mitochondrial fragmentation, Mdivi‐1 reduces ROS production, decreases mPTP opening, and alleviates oxidative stress [76]. Additionally, Mdivi‐1 has been shown to reduce infarct size and promote neurological recovery in ischemia–reperfusion injury models [77].

However, despite these promising findings, clinical use of Mdivi‐1 is limited by concerns over its specificity and potential off‐target effects. While inhibiting Drp1 can reduce mitochondrial fragmentation and cell death, it may also disrupt essential mitochondrial functions, such as energy metabolism, calcium buffering, and mitophagy. Long‐term Drp1 inhibition could result in mitochondrial dysfunction, metabolic disturbances, and compromised cellular homeostasis, leading to adverse effects on organ function and overall health. Therefore, potential off‐target and long‐term effects must be carefully considered in the development of Drp1‐targeted therapies [78].

Recent research has shifted toward the development of next‐generation Drp1 inhibitors with improved pharmacokinetic properties, enhanced selectivity, and reduced side effects [79]. While Mdivi‐1 has paved the way for Drp1‐targeted therapies, emerging compounds such as Dynasore, P110, and Drpitor1 offer promising new therapeutic avenues for further investigation. Table 1 summarizes a comparison of the major Drp1 inhibitors currently under investigation, including Dynasore, P110, and Drpitor1. Dynasore, which inhibits Drp1 and other GTPases, has been shown to reduce mitochondrial fragmentation, but its lack of specificity for Drp1 limits its clinical application [80]. The broad action of Dynasore introduces the potential for off‐target effects, making it unsuitable for Drp1‐specific therapy. P110, a peptide‐based inhibitor, selectively targets the Drp1‐Fis1 interaction and has demonstrated promising results in preclinical models, preserving mitochondrial function and reducing neuroinflammation. However, the peptide nature of P110 presents challenges in drug delivery, stability, and oral bioavailability, limiting its potential for clinical use without further optimization [81]. Drpitor1, a recently identified small molecule, selectively modulates Drp1 activity through PTMs such as Ser616 phosphorylation and Lys680 SUMOylation. Drpitor1 has shown high specificity and efficacy in reducing Drp1 overactivation, making it a promising candidate for therapeutic use in CIRI. However, further clinical validation is required to confirm its therapeutic potential and optimize its pharmacokinetic properties [82].

**TABLE 1:** Comparison of Drp1 Inhibitors (Dynasore, P110, Drpitor1) The table summarizes the mechanisms of action, advantages, limitations, and clinical readiness of these compounds.

Comparison of Drp1 inhibitors				
Compound	Mechanism of action	Advantages	Limitations	Clinical readiness
Dynasore	Inhibits Drp1 and other dynamin‐related GTPases	Effective in reducing mitochondrial fragmentation in ischemic models	Lack of specificity for Drp1; Affects other GTPases, leading to potential off‐target effects [80]	Limited clinical applicability due to non‐specificity [80]
P110	Selectively targets the Drp1‐Fis1 interaction	High specificity for Drp1; Preserves mitochondrial function; Reduces neuroinflammation [81]	Peptide‐based, leading to challenges in drug delivery stability, and oral bioavailability [81]	Potential for clinical use, but requires optimization for stability and delivery [81]
Drpitor1	Selectively modulates Drp1 activity through PTMs (e.g., Ser616 phosphorylation)	High specificity in reducing Drp1 overactivation; Promising for therapeutic use in CIRI [82]	Needs further clinical validation, optimization of pharmacokinetic properties required [82]	Strong potential for clinical use in CIRI, but requires additional clinical trials [82]

In addition to traditional small‐molecule inhibitors, recent advancements have focused on modulating Drp1's PTMs to achieve more precise control over its activity during CIRI. Specifically, Ser616 phosphorylation inhibitors can limit Drp1 overactivation, reducing mitochondrial fragmentation and inflammation [83]. These PTM‐targeting inhibitors offer a promising strategy for more context‐dependent modulation of Drp1, potentially improving therapeutic outcomes in diseases such as CIRI [84].

### 5.2 Gene Intervention Strategies

Gene‐based interventions provide a promising avenue for specifically regulating Drp1 expression and activity in the context of CIRI. RNA interference (RNAi) approaches, including small interfering RNA and short hairpin RNA, have been widely applied to knock down Drp1 expression, thereby reducing mitochondrial fission and limiting neuronal apoptosis [85]. CRISPR‐Cas9 gene editing further enables precise and durable modulation by allowing selective knockout or correction of Drp1 gene regions. This technique has shown sustained neuroprotective effects in both acute and chronic ischemia models [86]. Recent studies have introduced single‐guide RNAs specifically targeting functional domains of the Drp1 gene, including exons encoding the GTPase domain or phosphorylation sites such as Ser616, achieving domain‐specific repression of pathological activation while preserving physiological mitochondrial dynamics [87].

Despite these advances, gene‐based therapies still face major translational challenges, including low delivery efficiency, potential off‐target editing, and ethical concerns [88, 89]. To address these limitations, emerging nanocarrier‐based platforms are being explored for delivering Drp1‐targeted RNAi or CRISPR tools. Lipid‐based nanoparticles and polymeric nanocarriers have demonstrated improved BBB penetration and reduced immunogenicity in experimental systems. These systems are currently being optimized for enhanced targeting efficiency, long‐term safety, and clinical scalability [90], making gene‐specific modulation of Drp1 a viable therapeutic strategy for future intervention in CIRI.

### 5.3 Combination Therapy Strategies

While single‐target Drp1 inhibition has demonstrated efficacy in reducing ischemia–reperfusion injury, combination therapies can further enhance therapeutic outcomes by concurrently addressing multiple pathological mechanisms. Recent studies have explored the synergistic effects of combining Drp1 inhibitors with antioxidants, anti‐inflammatory agents, and mitochondrial protectants. For example, the combination of Drp1 inhibitors with antioxidants such as N‐acetylcysteine, vitamin E, or coenzyme Q10 has been shown to reduce ROS levels, prevent excessive mitochondrial fragmentation, and suppress neuronal apoptosis [91]. Moreover, co‐administering anti‐inflammatory agents with Drp1 inhibitors has shown potential in mitigating inflammation‐induced mitochondrial dysfunction and cell death. Additionally, mitochondrial protectants such as pyrroloquinoline quinone (PQQ) or CoQ10 can stabilize mitochondrial membrane potential, restore ATP production, and prevent oxidative stress‐induced damage [92, 93]. More recently, combination therapies specifically targeting Drp1's role in calcium overload and NLRP3 inflammasome activation have gained attention. For instance, pairing Drp1 inhibitors with calcium chelators or caspase‐1/NLRP3 inhibitors has demonstrated synergistic neuroprotection by simultaneously interrupting Drp1‐mediated mitochondrial fission and downstream inflammatory signaling pathways [94].

Furthermore, optimizing the dosage, route, and timing of multi‐agent administration remains a critical focus for enhancing efficacy and minimizing toxicity [95, 96]. Emerging nanomedicine platforms—including lipid nanoparticles and polymer‐based carriers—have shown promise in co‐delivering Drp1‐targeted compounds alongside anti‐inflammatory or antioxidant agents across the BBB. These nano‐formulations can be designed for controlled release, BBB targeting, and reduced immune activation, thereby improving brain‐specific drug delivery. Continued research into the safety, stability, and scalability of these delivery systems is essential for advancing combination therapies from bench to bedside in CIRI [97].

## 6 Conclusion and Prospect

### 6.1 Conclusion

Drp1 plays a central role in the pathogenesis of CIRI by orchestrating key pathological processes, including mitochondrial fission, oxidative stress, apoptosis, calcium dysregulation, and inflammation. Its overactivation leads to mitochondrial fragmentation, loss of ΔΨm, impaired ATP production, and the release of pro‐apoptotic factors, ultimately contributing to neuronal injury and neurological deficits. Thus, targeting Drp1 has emerged as a promising therapeutic strategy for improving outcomes in CIRI.

Pharmacological inhibitors such as Mdivi‐1 have shown neuroprotective potential by stabilizing mitochondrial function, reducing oxidative stress, and restoring energy metabolism in preclinical models. However, current inhibitors face limitations in specificity and long‐term safety, which hinder their clinical translation. Gene‐based interventions, including RNAi and CRISPR‐Cas9, offer more precise and sustained modulation of Drp1 expression and activity. Moreover, combination therapies that integrate Drp1 inhibitors with antioxidants, anti‐inflammatory agents, or mitochondrial stabilizers provide a multi‐targeted approach to interrupting multiple CIRI‐related cascades.

Despite these advances, several key challenges remain. Enhancing the target specificity of Drp1 inhibitors—particularly those directed at its GTPase domain or PTM sites—is essential to reduce off‐target effects. The BBB remains a major obstacle to effective drug delivery, necessitating the development of innovative delivery systems. In parallel, gene‐based approaches require further optimization of delivery vectors and minimization of off‐target risks to ensure safety and clinical feasibility.

Moreover, this review has several limitations that warrant acknowledgment. First, most of the evidence presented is based on rodent studies, which may not fully reflect human pathophysiology. Second, many therapeutic strategies—especially novel small molecules and gene‐editing tools—are still at the preclinical or early translational stage, requiring further validation. Third, due to Drp1's involvement in diverse cellular processes, isolating its specific role in CIRI without overlapping pathways remains difficult. Finally, the long‐term physiological consequences of sustained Drp1 inhibition remain unclear and must be cautiously evaluated in future research.

### 6.2 Prospect

Future research should focus on developing next‐generation Drp1 inhibitors with enhanced specificity, improved pharmacokinetic properties, and targeted regulation of PTMs. Structure‐based drug design and high‐throughput screening can facilitate the discovery of compounds that selectively bind to the Drp1 GTPase domain, adaptor protein interfaces, or regulatory sites such as Ser616 and Lys680. Inhibiting Ser616 phosphorylation or promoting SUMOylation at Lys680 may help suppress pathological Drp1 activation in CIRI. At the same time, gene‐editing tools like CRISPR‐Cas9 offer precise, long‐lasting modulation of Drp1 expression, especially when paired with optimized nanoparticle‐based delivery systems that improve BBB penetration and minimize off‐target effects.

A deeper understanding of Drp1's interaction with other pathological pathways—such as calcium dysregulation, inflammasome activation, and mitophagy—will enable the development of multimodal therapeutic strategies that more effectively address the complexity of CIRI. In parallel, identifying reliable biomarkers of Drp1 activity, including ΔΨm, mitochondrial fragmentation, or cytochrome c release, will be essential for monitoring treatment response. Advanced imaging technologies and omics‐based approaches, such as proteomics and metabolomics, may support patient stratification and the implementation of personalized treatment strategies.

Future studies should also evaluate the therapeutic potential of Drp1‐targeted strategies in comorbid conditions frequently associated with CIRI, such as diabetes, hypertension, and chronic inflammation. These conditions may influence Drp1 signaling or mitochondrial vulnerability, thus affecting treatment outcomes. Given Drp1's multifaceted role in both physiological and pathological processes, all therapeutic strategies must undergo rigorous safety assessments to avoid off‐target consequences. Progress in these areas will be critical for establishing Drp1‐targeted interventions as a cornerstone in the management of ischemia–reperfusion injury and related neurological disorders.

## Ethics Statement

This review paper adheres to ethical guidelines and standards set forth by relevant academic and scientific communities. The authors declare that the research discussed in this review complies with the ethical principles of the Declaration of Helsinki and other applicable ethical frameworks.

## Consent

All authors agree with the content of the manuscript.

## Conflicts of Interest

The authors declare no conflicts of interest.

## Data Availability

The data and information used in this review article are sourced from publicly available, published literature. All data referenced in the article can be accessed through the respective sources cited. No new experimental data were generated for this study, and as such, there is no associated data storage or sharing. For further details on specific data or methodologies, readers are encouraged to consult the cited references or contact the corresponding author for additional information.

## References

[1] M. Y. Wu , G. T. Yiang , W. T. Liao , et al., “Current Mechanistic Concepts in Ischemia and Reperfusion Injury,” Cellular Physiology and Biochemistry 46, no. 4 (2018): 1650–1667. 29694958 10.1159/000489241

[2] G. M. Fogo , S. Raghunayakula , K. J. Emaus , et al., “Mitochondrial Dynamics and Quality Control Regulate Proteostasis in Neuronal Ischemia‐Reperfusion,” Autophagy 16 (2025): 1–15. 10.1080/15548627.2025.2472586 PMC12283014 40016670

[3] X. Ma , Y. Xie , Y. Chen , B. Han , J. Li , and S. Qi , “Post‐Ischemia Mdivi‐1 Treatment Protects Against Ischemia/Reperfusion‐Induced Brain Injury in a Rat Model,” Neuroscience Letters 632 (2016): 23–32. 27542342 10.1016/j.neulet.2016.08.026

[4] K. Atkins , A. Dasgupta , K. H. Chen , J. Mewburn , and S. L. Archer , “The Role of Drp1 Adaptor Proteins MiD49 and MiD51 in Mitochondrial Fission: Implications for Human Disease,” Clinical Science (London, England) 130, no. 21 (2016): 1861–1874. 10.1042/CS20160030 27660309

[5] S. Orellana‐Urzúa , I. Rojas , L. Líbano , and R. Rodrigo , “Pathophysiology of Ischemic Stroke: Role of Oxidative Stress,” Current Pharmaceutical Design 26, no. 34 (2020): 4246–4260. 32640953 10.2174/1381612826666200708133912

[6] J. Huang , L. Chen , Z. M. Yao , X. R. Sun , X. H. Tong , and S. Y. Dong , “The Role of Mitochondrial Dynamics in Cerebral Ischemia‐Reperfusion Injury,” Biomedicine & Pharmacotherapy 162 (2023): 114671. 37037094 10.1016/j.biopha.2023.114671

[7] A. P. Halestrap , “Calcium, Mitochondria and Reperfusion Injury: A Pore Way to Die,” Biochemical Society Transactions 34, no. Pt 2 (2006): 232–237. 16545083 10.1042/BST20060232

[8] L. Shen , Q. Gan , Y. Yang , et al., “Mitophagy in Cerebral Ischemia and Ischemia/Reperfusion Injury,” Frontiers in Aging Neuroscience 13 (2021): 687246. 34168551 10.3389/fnagi.2021.687246 PMC8217453

[9] P. P. Zhu , A. Patterson , J. Stadler , D. P. Seeburg , M. Sheng , and C. Blackstone , “Intra‐ and Intermolecular Domain Interactions of the C‐Terminal GTPase Effector Domain of the Multimeric Dynamin‐Like GTPase Drp1,” Journal of Biological Chemistry 279, no. 34 (2004): 35967–35974. 15208300 10.1074/jbc.M404105200

[10] N. Taguchi , N. Ishihara , A. Jofuku , T. Oka , and K. Mihara , “Mitotic Phosphorylation of Dynamin‐Related GTPase Drp1 Participates in Mitochondrial Fission,” Journal of Biological Chemistry 282, no. 15 (2007): 11521–11529. 17301055 10.1074/jbc.M607279200

[11] B. L. Bauer , K. Rochon , J. C. Liu , R. Ramachandran , and J. A. Mears , “Disease‐Associated Mutations in Drp1 Have Fundamentally Different Effects on the Mitochondrial Fission Machinery,” Human Molecular Genetics 32, no. 12 (2023): 1975–1987. 36795043 10.1093/hmg/ddad029 PMC10244223

[12] K. R. Pitts , M. A. McNiven , and Y. Yoon , “Mitochondria‐Specific Function of the Dynamin Family Protein DLP1 Is Mediated by Its C‐Terminal Domains,” Journal of Biological Chemistry 279, no. 48 (2004): 50286–50294. 15364948 10.1074/jbc.M405531200

[13] L. Tilokani , S. Nagashima , V. Paupe , and J. Prudent , “Mitochondrial Dynamics: Overview of Molecular Mechanisms,” Essays in Biochemistry 62, no. 3 (2018): 341–360. 30030364 10.1042/EBC20170104 PMC6056715

[14] X. Chen , T. Yang , Y. Zhou , Z. Mei , and W. Zhang , “Astragaloside IV Combined With Ligustrazine Ameliorates Abnormal Mitochondrial Dynamics via Drp1 SUMO/deSUMOylation in Cerebral Ischemia‐Reperfusion Injury,” CNS Neuroscience & Therapeutics 30, no. 4 (2024): e14725. 38615367 10.1111/cns.14725 PMC11016344

[15] E. Napoli , G. Song , S. Liu , et al., “Zdhhc13‐Dependent Drp1 S‐Palmitoylation Impacts Brain Bioenergetics, Anxiety, Coordination and Motor Skills,” Scientific Reports 7, no. 1 (2017): 12796. 29038583 10.1038/s41598-017-12889-0 PMC5643561

[16] Q. Hu , H. Zhang , N. Gutiérrez Cortés , et al., “Increased Drp1 Acetylation by Lipid Overload Induces Cardiomyocyte Death and Heart Dysfunction,” Circulation Research 126, no. 4 (2020): 456–470. 31896304 10.1161/CIRCRESAHA.119.315252 PMC7035202

[17] K. Schmitt , A. Grimm , R. Dallmann , et al., “Circadian Control of DRP1 Activity Regulates Mitochondrial Dynamics and Bioenergetics,” Cell Metabolism 27, no. 3 (2018): 657–666.e5. 29478834 10.1016/j.cmet.2018.01.011

[18] X. Yu , L. Jia , W. Yu , and H. du , “Dephosphorylation by Calcineurin Regulates Translocation of Dynamin‐Related Protein 1 to Mitochondria in Hepatic Ischemia Reperfusion Induced Hippocampus Injury in Young Mice,” Brain Research 1711 (2019): 68–76. 30659828 10.1016/j.brainres.2019.01.018

[19] X. Chang , S. Niu , M. Shang , et al., “ROS‐Drp1‐Mediated Mitochondria Fission Contributes to Hippocampal HT22 Cell Apoptosis Induced by Silver Nanoparticles,” Redox Biology 63 (2023): 102739. 37187014 10.1016/j.redox.2023.102739 PMC10199224

[20] A. Peña‐Blanco and A. J. García‐Sáez , “Bax, Bak and Beyond ‐ Mitochondrial Performance in Apoptosis,” FEBS Journal 285, no. 3 (2018): 416–431. 28755482 10.1111/febs.14186

[21] Q. Wu , J. Liu , Z. Mao , et al., “Ligustilide Attenuates Ischemic Stroke Injury by Promoting Drp1‐Mediated Mitochondrial Fission via Activation of AMPK,” Phytomedicine 95 (2022): 153884. 34929562 10.1016/j.phymed.2021.153884

[22] V. H. Sathyamurthy , Y. Nagarajan , and V. D. Parvathi , “Mitochondria‐Endoplasmic Reticulum Contact Sites (MERCS): A New Axis in Neuronal Degeneration and Regeneration,” Molecular Neurobiology 61, no. 9 (2024): 6528–6538. 38321352 10.1007/s12035-024-03971-6

[23] H. Otera , N. Ishihara , and K. Mihara , “New Insights Into the Function and Regulation of Mitochondrial Fission,” Biochimica et Biophysica Acta 1833, no. 5 (2013): 1256–1268. 23434681 10.1016/j.bbamcr.2013.02.002

[24] M. T. Breitzig , M. D. Alleyn , R. F. Lockey , and N. Kolliputi , “A Mitochondrial Delicacy: Dynamin‐Related Protein 1 and Mitochondrial Dynamics,” American Journal of Physiology. Cell Physiology 315, no. 1 (2018): C80–C90. 29669222 10.1152/ajpcell.00042.2018 PMC6087727

[25] A. R. Anzell , G. M. Fogo , Z. Gurm , et al., “Mitochondrial Fission and Mitophagy Are Independent Mechanisms Regulating Ischemia/Reperfusion Injury in Primary Neurons,” Cell Death & Disease 12, no. 5 (2021): 475. 33980811 10.1038/s41419-021-03752-2 PMC8115279

[26] M. Zerihun , S. Sukumaran , and N. Qvit , “The Drp1‐Mediated Mitochondrial Fission Protein Interactome as an Emerging Core Player in Mitochondrial Dynamics and Cardiovascular Disease Therapy,” International Journal of Molecular Sciences 24, no. 6 (2023): 5785. 36982862 10.3390/ijms24065785 PMC10057413

[27] R. K. Dagda , S. J. Cherra, 3rd , S. M. Kulich , A. Tandon , D. Park , and C. T. Chu , “Loss of PINK1 Function Promotes Mitophagy Through Effects on Oxidative Stress and Mitochondrial Fission,” Journal of Biological Chemistry 284, no. 20 (2009): 13843–13855. 19279012 10.1074/jbc.M808515200 PMC2679485

[28] P. R. Haynes , E. S. Pyfrom , Y. Li , et al., “A Neuron‐Glia Lipid Metabolic Cycle Couples Daily Sleep to Mitochondrial Homeostasis,” Nature Neuroscience 27, no. 4 (2024): 666–678. 38360946 10.1038/s41593-023-01568-1 PMC11001586

[29] X. Gu , W. Chen , Z. Li , X. Wang , Q. Su , and F. Zhou , “Drp1 Mitochondrial Fission in Astrocyte Modulates Behavior and Neuroinflammation During Morphine Addiction,” Journal of Neuroinflammation 22, no. 1 (2025): 108. 40247294 10.1186/s12974-025-03438-y PMC12007278

[30] Y. J. Yuan , T. Chen , Y. L. Yang , H. N. Han , and L. M. Xu , “E2F1/CDK5/DRP1 Axis Mediates Microglial Mitochondrial Division and Autophagy in the Pathogenesis of Cerebral Ischemia‐Reperfusion Injury,” Clinical and Translational Medicine 15, no. 2 (2025): e70197. 39968698 10.1002/ctm2.70197 PMC11836619

[31] C. Hu , Y. Huang , and L. Li , “Drp1‐Dependent Mitochondrial Fission Plays Critical Roles in Physiological and Pathological Progresses in Mammals,” International Journal of Molecular Sciences 18, no. 1 (2017): 144. 28098754 10.3390/ijms18010144 PMC5297777

[32] T. Kalogeris , C. P. Baines , M. Krenz , and R. J. Korthuis , “Cell Biology of Ischemia/Reperfusion Injury,” International Review of Cell and Molecular Biology 298 (2012): 229–317. 22878108 10.1016/B978-0-12-394309-5.00006-7 PMC3904795

[33] N. M. B. Yapa , V. Lisnyak , B. Reljic , and M. T. Ryan , “Mitochondrial Dynamics in Health and Disease,” FEBS Letters 595, no. 8 (2021): 1184–1204. 33742459 10.1002/1873-3468.14077

[34] J. M. Quiles and Å. B. Gustafsson , “The Role of Mitochondrial Fission in Cardiovascular Health and Disease,” Nature Reviews. Cardiology 19, no. 11 (2022): 723–736. 35523864 10.1038/s41569-022-00703-y PMC10584015

[35] A. M. Bertholet , T. Delerue , A. M. Millet , et al., “Mitochondrial Fusion/Fission Dynamics in Neurodegeneration and Neuronal Plasticity,” Neurobiology of Disease 90 (2016): 3–19. 26494254 10.1016/j.nbd.2015.10.011

[36] H. Du , Y. He , J. Zhu , et al., “Danhong Injection Alleviates Cerebral Ischemia‐Reperfusion Injury by Inhibiting Mitochondria‐Dependent Apoptosis Pathway and Improving Mitochondrial Function in Hyperlipidemia Rats,” Biomedicine & Pharmacotherapy 157 (2023): 114075. 36481401 10.1016/j.biopha.2022.114075

[37] X. C. Ni , H. F. Wang , Y. Y. Cai , et al., “Ginsenoside Rb1 Inhibits Astrocyte Activation and Promotes Transfer of Astrocytic Mitochondria to Neurons Against Ischemic Stroke,” Redox Biology 54 (2022): 102363. 35696763 10.1016/j.redox.2022.102363 PMC9198466

[38] M. Wu , X. Gu , and Z. Ma , “Mitochondrial Quality Control in Cerebral Ischemia‐Reperfusion Injury,” Molecular Neurobiology 58, no. 10 (2021): 5253–5271. 34275087 10.1007/s12035-021-02494-8

[39] L. C. Tábara , M. Segawa , and J. Prudent , “Molecular Mechanisms of Mitochondrial Dynamics,” Nature Reviews. Molecular Cell Biology 26, no. 2 (2025): 123–146. 39420231 10.1038/s41580-024-00785-1

[40] Y. Zhang and X. Gong , “Fat Mass and Obesity Associated Protein Inhibits Neuronal Ferroptosis via the FYN/Drp1 Axis and Alleviate Cerebral Ischemia/Reperfusion Injury,” CNS Neuroscience & Therapeutics 30, no. 3 (2024): e14636. 38430221 10.1111/cns.14636 PMC10908355

[41] N. Xie , C. Wang , Y. Lian , H. Zhang , C. Wu , and Q. Zhang , “A Selective Inhibitor of Drp1, Mdivi‐1, Protects Against Cell Death of Hippocampal Neurons in Pilocarpine‐Induced Seizures in Rats,” Neuroscience Letters 545 (2013): 64–68. 23628672 10.1016/j.neulet.2013.04.026

[42] T. Yu , L. Wang , L. Zhang , and P. A. Deuster , “Mitochondrial Fission as a Therapeutic Target for Metabolic Diseases: Insights Into Antioxidant Strategies,” Antioxidants (Basel) 12, no. 6 (2023): 1163. 37371893 10.3390/antiox12061163 PMC10295595

[43] L. Zhou , Q. Zhang , P. Zhang , et al., “C‐Abl‐Mediated Drp1 Phosphorylation Promotes Oxidative Stress‐Induced Mitochondrial Fragmentation and Neuronal Cell Death,” Cell Death & Disease 8, no. 10 (2017): e3117. 29022905 10.1038/cddis.2017.524 PMC5682686

[44] T. D. Fischer , M. J. Hylin , J. Zhao , et al., “Altered Mitochondrial Dynamics and TBI Pathophysiology,” Frontiers in Systems Neuroscience 10 (2016): 29. 27065821 10.3389/fnsys.2016.00029 PMC4811888

[45] D. Chen , H. Duan , C. Zou , et al., “20(R)‐Ginsenoside Rg3 Attenuates Cerebral Ischemia‐Reperfusion Injury by Mitigating Mitochondrial Oxidative Stress via the Nrf2/HO‐1 Signaling Pathway,” Phytotherapy Research 38, no. 3 (2024): 1462–1477. 38246696 10.1002/ptr.8118

[46] Y. Li , H. Chen , Q. Yang , et al., “Increased Drp1 Promotes Autophagy and ESCC Progression by mtDNA Stress Mediated cGAS‐STING Pathway,” Journal of Experimental & Clinical Cancer Research 41, no. 1 (2022): 76. 35209954 10.1186/s13046-022-02262-z PMC8867650

[47] M. Zhang , Q. Liu , H. Meng , et al., “Ischemia‐Reperfusion Injury: Molecular Mechanisms and Therapeutic Targets,” Signal Transduction and Targeted Therapy 9, no. 1 (2024): 12. 38185705 10.1038/s41392-023-01688-x PMC10772178

[48] D. Yuan , C. Liu , and B. Hu , “Dysfunction of Membrane Trafficking Leads to Ischemia‐Reperfusion Injury After Transient Cerebral Ischemia,” Translational Stroke Research 9, no. 3 (2018): 215–222. 29022237 10.1007/s12975-017-0572-0 PMC5895539

[49] D. Arnoult , N. Rismanchi , A. Grodet , et al., “Bax/Bak‐Dependent Release of DDP/TIMM8a Promotes Drp1‐Mediated Mitochondrial Fission and Mitoptosis During Programmed Cell Death,” Current Biology 15, no. 23 (2005): 2112–2118. 16332536 10.1016/j.cub.2005.10.041

[50] P. A. Parone , D. I. James , S. Da Cruz , et al., “Inhibiting the Mitochondrial Fission Machinery Does Not Prevent Bax/Bak‐Dependent Apoptosis,” Molecular and Cellular Biology 26, no. 20 (2006): 7397–7408. 17015472 10.1128/MCB.02282-05 PMC1636857

[51] Y. Tang , X. Liu , J. Zhao , et al., “Hypothermia‐Induced Ischemic Tolerance Is Associated With Drp1 Inhibition in Cerebral Ischemia‐Reperfusion Injury of Mice,” Brain Research 1646 (2016): 73–83. 27235868 10.1016/j.brainres.2016.05.042

[52] H. Wang , S. Zheng , M. Liu , et al., “The Effect of Propofol on Mitochondrial Fission During Oxygen‐Glucose Deprivation and Reperfusion Injury in Rat Hippocampal Neurons,” PLoS One 11, no. 10 (2016): e0165052. 27788177 10.1371/journal.pone.0165052 PMC5082830

[53] L. Delavallée , L. Cabon , P. Galán‐Malo , H. K. Lorenzo , and S. A. Susin , “AIF‐Mediated Caspase‐Independent Necroptosis: A New Chance for Targeted Therapeutics,” IUBMB Life 63, no. 4 (2011): 221–232. 21438113 10.1002/iub.432

[54] B. C. White , J. M. Sullivan , D. J. DeGracia , et al., “Brain Ischemia and Reperfusion: Molecular Mechanisms of Neuronal Injury,” Journal of the Neurological Sciences 179, no. S 1‐2 (2000): 1–33. 11054482 10.1016/s0022-510x(00)00386-5

[55] D. G. Lee , J. S. Min , H. S. Lee , and D. S. Lee , “Isoliquiritigenin Attenuates Glutamate‐Induced Mitochondrial Fission via Calcineurin‐Mediated Drp1 Dephosphorylation in HT22 Hippocampal Neuron Cells,” Neurotoxicology 68 (2018): 133–141. 30048666 10.1016/j.neuro.2018.07.011

[56] L. Piao , Y. H. Fang , M. Fisher , et al., “Dynamin‐Related Protein 1 Is a Critical Regulator of Mitochondrial Calcium Homeostasis During Myocardial Ischemia/Reperfusion Injury,” FASEB Journal 38, no. 1 (2024): e23379. 38133921 10.1096/fj.202301040RR PMC12027350

[57] A. Ruiz , E. Alberdi , and C. Matute , “Mitochondrial Division Inhibitor 1 (Mdivi‐1) Protects Neurons Against Excitotoxicity Through the Modulation of Mitochondrial Function and Intracellular Ca2+ Signaling,” Frontiers in Molecular Neuroscience 11, (2018): 3. 29386996 10.3389/fnmol.2018.00003 PMC5776080

[58] J. Guo , L. Zhang , Y. Bu , W. Li , J. Hu , and J. Li , “Ras‐Related Protein Rab‐20 Inhibition Alleviates Cerebral Ischemia/Reperfusion Injury by Inhibiting Mitochondrial Fission and Dysfunction,” Frontiers in Molecular Neuroscience 15 (2022): 986710. 36385754 10.3389/fnmol.2022.986710 PMC9640763

[59] D. J. Fawthrop , A. R. Boobis , and D. S. Davies , “Mechanisms of Cell Death,” Archives of Toxicology 65, no. 6 (1991): 437–444. 1929863 10.1007/BF01977355

[60] K. H. Flippo , A. Gnanasekaran , G. A. Perkins , et al., “AKAP1 Protects From Cerebral Ischemic Stroke by Inhibiting Drp1‐Dependent Mitochondrial Fission,” Journal of Neuroscience 38, no. 38 (2018): 8233–8242. 30093535 10.1523/JNEUROSCI.0649-18.2018 PMC6146498

[61] T. Suetomi , A. Willeford , C. S. Brand , et al., “Inflammation and NLRP3 Inflammasome Activation Initiated in Response to Pressure Overload by Ca2+/Calmodulin‐Dependent Protein Kinase II δ Signaling in Cardiomyocytes Are Essential for Adverse Cardiac Remodeling,” Circulation 138, no. 22 (2018): 2530–2544. 30571348 10.1161/CIRCULATIONAHA.118.034621 PMC6309790

[62] Q. Jiang , Y. Ding , F. Li , A. I. Fayyaz , H. Duan , and X. Geng , “Modulation of NLRP3 Inflammasome‐Related‐Inflammation via RIPK1/RIPK3‐DRP1 or HIF‐1α Signaling by Phenothiazine in Hypothermic and Normothermic Neuroprotection After Acute Ischemic Stroke,” Redox Biology 73 (2024): 103169. 38692093 10.1016/j.redox.2024.103169 PMC11070764

[63] X. Zeng , Y. D. Zhang , R. Y. Ma , et al., “Activated Drp1 Regulates p62‐Mediated Autophagic Flux and Aggravates Inflammation in Cerebral Ischemia‐Reperfusion via the ROS‐RIP1/RIP3‐Exosome Axis,” Military Medical Research 9, no. 1 (2022): 25. 35624495 10.1186/s40779-022-00383-2 PMC9137164

[64] R. Liu , S. C. Wang , M. Li , et al., “Erratum to ‘An Inhibitor of DRP1 (Mdivi‐1) Alleviates LPS‐Induced Septic AKI by Inhibiting NLRP3 Inflammasome Activation’,” BioMed Research International 2020 (2020): 8493938. 33015183 10.1155/2020/8493938 PMC7520697

[65] R. Q. Yao , C. Ren , Z. F. Xia , and Y. M. Yao , “Organelle‐Specific Autophagy in Inflammatory Diseases: A Potential Therapeutic Target Underlying the Quality Control of Multiple Organelles,” Autophagy 17, no. 2 (2021): 385–401. 32048886 10.1080/15548627.2020.1725377 PMC8007140

[66] Z. D. Su , C. Q. Li , H. W. Wang , M. M. Zheng , and Q. W. Chen , “Inhibition of DRP1‐Dependent Mitochondrial Fission by Mdivi‐1 Alleviates Atherosclerosis Through the Modulation of M1 Polarization,” Journal of Translational Medicine 21, no. 1 (2023): 427. 37386574 10.1186/s12967-023-04270-9 PMC10311781

[67] P. K. Nandan , A. T. Job , and T. Ramasamy , “DRP1 Association in Inflammation and Metastasis: A Review,” Current Drug Targets 25, no. 13 (2024): 909–918. 39248071 10.2174/0113894501304751240819111831

[68] W. L. Hong , H. Huang , X. Zeng , and C. Y. Duan , “Targeting Mitochondrial Quality Control: New Therapeutic Strategies for Major Diseases,” Military Medical Research 11, no. 1 (2024): 59. 39164792 10.1186/s40779-024-00556-1 PMC11337860

[69] Q. Q. Zhang , L. Luo , M. X. Liu , et al., “Enriched Environment‐Induced Neuroprotection Against Cerebral Ischemia‐Reperfusion Injury Might be Mediated via Enhancing Autophagy Flux and Mitophagy Flux,” Mediators of Inflammation 2022 (2022): 2396487. 35795405 10.1155/2022/2396487 PMC9252718

[70] K. H. Flippo and S. Strack , “Mitochondrial Dynamics in Neuronal Injury, Development and Plasticity,” Journal of Cell Science 130, no. 4 (2017): 671–681. 28154157 10.1242/jcs.171017 PMC5339882

[71] Y. Zhang , Y. He , M. Wu , et al., “Rehmapicroside Ameliorates Cerebral Ischemia‐Reperfusion Injury via Attenuating Peroxynitrite‐Mediated Mitophagy Activation,” Free Radical Biology & Medicine 160 (2020): 526–539. 32784031 10.1016/j.freeradbiomed.2020.06.034

[72] S. Hao , H. Huang , R. Y. Ma , X. Zeng , and C. Y. Duan , “Multifaceted Functions of Drp1 in Hypoxia/Ischemia‐Induced Mitochondrial Quality Imbalance: From Regulatory Mechanism to Targeted Therapeutic Strategy,” Military Medical Research 10, no. 1 (2023): 46. 37833768 10.1186/s40779-023-00482-8 PMC10571487

[73] Y. Huan , G. Hao , Z. Shi , Y. Liang , Y. Dong , and H. Quan , “The Role of Dynamin‐Related Protein 1 in Cerebral Ischemia/Hypoxia Injury,” Biomedicine & Pharmacotherapy 165 (2023): 115247. 37516018 10.1016/j.biopha.2023.115247

[74] K. Ma , G. Chen , W. Li , O. Kepp , Y. Zhu , and Q. Chen , “Mitophagy, Mitochondrial Homeostasis, and Cell Fate,” Frontiers in Cell and Development Biology 8 (2020): 467. 10.3389/fcell.2020.00467 PMC7326955 32671064

[75] E. A. Bordt , P. A. Clerc , B. A. Roelofs , et al., “The Putative Drp1 Inhibitor Mdivi‐1 Is a Reversible Mitochondrial Complex I Inhibitor That Modulates Reactive Oxygen Species,” Developmental Cell 40 (2017): 583–594. 28350990 10.1016/j.devcel.2017.02.020 PMC5398851

[76] C. Duan , L. Wang , J. Zhang , et al., “Mdivi‐1 Attenuates Oxidative Stress and Exerts Vascular Protection in Ischemic/Hypoxic Injury by a Mechanism Independent of Drp1 GTPase Activity,” Redox Biology 37 (2020): 101706. 32911435 10.1016/j.redox.2020.101706 PMC7490562

[77] N. T. Nhu , Q. Li , Y. Liu , J. Xu , S. Y. Xiao , and S. D. Lee , “Effects of Mdivi‐1 on Neural Mitochondrial Dysfunction and Mitochondria‐Mediated Apoptosis in Ischemia‐Reperfusion Injury After Stroke: A Systematic Review of Preclinical Studies,” Frontiers in Molecular Neuroscience 14 (2021): 778569. 35002619 10.3389/fnmol.2021.778569 PMC8740201

[78] G. Smith and G. Gallo , “To Mdivi‐1 or Not to Mdivi‐1: Is That the Question?,” Developmental Neurobiology 77, no. 11 (2017): 1260–1268. 28842943 10.1002/dneu.22519 PMC5654677

[79] A. A. Rosdah , B. M. Abbott , C. G. Langendorf , et al., “A Novel Small Molecule Inhibitor of Human Drp1,” Scientific Reports 12, no. 1 (2022): 21531. 36513726 10.1038/s41598-022-25464-z PMC9747717

[80] E. Macia , M. Ehrlich , R. Massol , E. Boucrot , C. Brunner , and T. Kirchhausen , “Dynasore, a Cell‐Permeable Inhibitor of Dynamin,” Developmental Cell 10, no. 6 (2006): 839–850. 16740485 10.1016/j.devcel.2006.04.002

[81] X. Qi , N. Qvit , Y. C. Su , and D. Mochly‐Rosen , “A Novel Drp1 Inhibitor Diminishes Aberrant Mitochondrial Fission and Neurotoxicity,” Journal of Cell Science 126, no. Pt 3 (2013): 789–802. 23239023 10.1242/jcs.114439 PMC3619809

[82] D. Wu , A. Dasgupta , K. H. Chen , et al., “Identification of Novel Dynamin‐Related Protein 1 (Drp1) GTPase Inhibitors: Therapeutic Potential of Drpitor1 and Drpitor1a in Cancer and Cardiac Ischemia‐Reperfusion Injury,” FASEB Journal 34, no. 1 (2020): 1447–1464. 31914641 10.1096/fj.201901467R

[83] H. Wen , X. Tu , F. Luo , et al., “A Novel PDE4 Inhibitor ZX21011 Alleviates Neuronal Apoptosis by Decreasing GSK3β‐Mediated Drp1 Ser616 Phosphorylation in Cerebral Ischemia Reperfusion,” Chemico‐Biological Interactions 408 (2025): 111405. 39889870 10.1016/j.cbi.2025.111405

[84] P. Das and O. Chakrabarti , “Publisher Correction: ISGylation of DRP1 Closely Balances Other Post‐Translational Modifications to Mediate Mitochondrial Fission,” Cell Death & Disease 15, no. 7 (2024): 488. 38982063 10.1038/s41419-024-06857-6 PMC11233681

[85] S. An , Y. Kuang , T. Shen , et al., “Brain‐Targeting Delivery for RNAi Neuroprotection Against Cerebral Ischemia Reperfusion Injury,” Biomaterials 34, no. 35 (2013): 8949–8959. 23968852 10.1016/j.biomaterials.2013.07.060

[86] Y. Ma , L. Zhang , and X. Huang , “Genome Modification by CRISPR/Cas9,” FEBS Journal 281, no. 23 (2014): 5186–5193. 25315507 10.1111/febs.13110

[87] E. E. Ellison , U. Nagalakshmi , M. E. Gamo , et al., “Multiplexed Heritable Gene Editing Using RNA Viruses and Mobile Single Guide RNAs,” Nature Plants 7, no. 1 (2021): 99. 33328598 10.1038/s41477-020-00837-2

[88] F. Zhang , Y. Wen , and X. Guo , “CRISPR/Cas9 for Genome Editing: Progress, Implications and Challenges,” Human Molecular Genetics 23, no. R1 (2014): R40–R46. 24651067 10.1093/hmg/ddu125

[89] S. N. Devi , R. Rana , P. Malik , and N. K. Ganguly , “CRISPR/Cas9: An Overview of Recent Developments and Applications in Cancer Research,” International Journal of Surgery 110, no. 10 (2024): 6198–6213. 38377059 10.1097/JS9.0000000000001081 PMC11486967

[90] D. C. Luther , Y. W. Lee , H. Nagaraj , F. Scaletti , and V. M. Rotello , “Delivery Approaches for CRISPR/Cas9 Therapeutics In Vivo: Advances and Challenges,” Expert Opinion on Drug Delivery 15, no. 9 (2018): 905–913. 30169977 10.1080/17425247.2018.1517746 PMC6295289

[91] P. H. Reddy , M. Manczak , X. Yin , and A. P. Reddy , “Synergistic Protective Effects of Mitochondrial Division Inhibitor 1 and Mitochondria‐Targeted Small Peptide SS31 in Alzheimer's Disease,” Journal of Alzheimer's Disease 62, no. 4 (2018): 1549–1565. 10.3233/JAD-170988 PMC5884714 29400667

[92] R. Troncoso , F. Paredes , V. Parra , et al., “Dexamethasone‐Induced Autophagy Mediates Muscle Atrophy Through Mitochondrial Clearance,” Cell Cycle 13, no. 14 (2014): 2281–2295. 24897381 10.4161/cc.29272 PMC4111682

[93] K. Takahashi , I. Ohsawa , T. Shirasawa , and M. Takahashi , “Optic Atrophy 1 Mediates Coenzyme Q‐Responsive Regulation of Respiratory Complex IV Activity in Brain Mitochondria,” Experimental Gerontology 98 (2017): 217–223. 28890359 10.1016/j.exger.2017.09.002

[94] J. Li , Y. Bu , B. Li , et al., “Calenduloside E Alleviates Cerebral Ischemia/Reperfusion Injury by Preserving Mitochondrial Function,” Journal of Molecular Histology 53, no. 4 (2022): 713–727. 35819738 10.1007/s10735-022-10087-5 PMC9374638

[95] H. Baghirov , “Receptor‐Mediated Transcytosis of Macromolecules Across the Blood‐Brain Barrier,” Expert Opinion on Drug Delivery 20, no. 12 (2023): 1699–1711. 37658673 10.1080/17425247.2023.2255138

[96] G. C. Terstappen , A. H. Meyer , R. D. Bell , and W. Zhang , “Strategies for Delivering Therapeutics Across the Blood‐Brain Barrier,” Nature Reviews. Drug Discovery 20, no. 5 (2021): 362–383. 33649582 10.1038/s41573-021-00139-y

[97] A. Lopalco , R. M. Iacobazzi , A. A. Lopedota , and N. Denora , “Recent Advances in Nanodrug Delivery Systems Production, Efficacy, Safety, and Toxicity,” Methods in Molecular Biology 2834 (2025): 303–332. 39312172 10.1007/978-1-0716-4003-6_15

